# The Effects of a Forest Therapy Programme on Mental Hospital Patients with Affective and Psychotic Disorders

**DOI:** 10.3390/ijerph17010118

**Published:** 2019-12-23

**Authors:** Ernest Bielinis, Aneta Jaroszewska, Adrian Łukowski, Norimasa Takayama

**Affiliations:** 1 Department of Forestry and Forest Ecology, Faculty of Environmental Management and Agriculture, University of Warmia and Mazury, Pl. Łódzki 2, 10-727 Olsztyn, Poland; 2 Department of Psychiatry, University of Warmia and Mazury, Aleja Wojska Polskiego 35, 11-041 Olsztyn, Poland; anetajaroszewska@tlen.pl; 3 Faculty of Forestry, Poznań University of Life Sciences, Wojska Polskiego 71c, 60-625 Poznań, Poland; adrian.lukowski@up.poznan.pl; 4 Environmental Planning Laboratory, Department of Forest Management, Forestry and Forest Products Research Institute in Japan, 1 Matsunosato, Tsukuba, Ibaraki 305-8687, Japan; hanri@ffpri.affrc.go.jp

**Keywords:** depression, forest bathing, forest therapy, mental disorder, mental hospital inpatients, psychosis, Shinrin-yoku

## Abstract

The positive effect of forest bathing on the mental health and wellbeing of those suffering from post-traumatic stress disorder or experiencing stress has been proven. It is not known, however, how ‘forest therapy’ affects the mental health of people who are treated in a psychiatric hospital for affective or psychotic disorders. Potentially, forest therapy could bring many benefits to these people. To test the potential effectiveness of this therapy, a quasi-experiment was carried out in a psychiatric hospital in Olsztyn (north Poland). In the summer and autumn of 2018, the patients of the psychiatric hospital in Olsztyn participated in forest therapy interventions. The proposed forest therapy consisted of participating in one hour and forty-five minutes walks under the supervision of a therapist. Subjects filled out the Profile of Mood States Questionnaire (POMS) and the State Trait Anxiety Inventory (STAI-S) before and after the study. In the case of a group of patients with affective disorders, forest therapy had a positive effect on nearly all POMS scale subscales, with the exception of the ‘anger–hostility’ subscale, which did not change its values significantly after the intervention. In these patients, the greatest impacts were noted in the subscales ‘confusion’ and ‘depression–dejection’; the level of anxiety measured with the STAI-S scale also significantly decreased. In the case of patients with psychotic disorders, the values of the ‘confusion’ and ‘vigour’ subscales and the STAI-S scale exhibited the greatest changes. These changes were positive for the health of patients. Regarding the ‘fatigue’ subscale, no significant changes were observed in patients with psychotic disorders. The observed changes in psychological indicators in psychiatric hospital patients with both kinds of disorders indicate that the intervention of forest therapy can positively affect their mental health. The changes observed in psychological indicators were related to the characteristics of the given disorder.

## 1. Introduction

Forest recreation is any activity conducted in a forest environment for pleasure and to refresh the mental attitude of an individual [1]. One type of forest recreation meant to improve human health is often called forest therapy, forest bathing or *Shinrin-yoku*, and is often used as an alternative method to treat many afflictions. Nevertheless, the effectiveness of forest recreation as a complementary therapy for mental hospital inpatients has not yet been examined with a large sample size. Several studies have confirmed that this therapy is effective for some psychiatric conditions, such as in the treatment of depression and anxiety in patients with chronic stroke [2], in the cure of depression [3,4], and as treatment for depression of alcoholics [5] and post-traumatic stress disorder [6]. The effect on a larger sample of patients (more than 20) in a mental hospital, however, has not been examined in any studies reported in the accessible literature (e.g., in [7] there were 20 patients involved, 10 per group).

Forest therapy is also helpful in coping with chronic widespread pain [8] and in lowering blood pressure in hypertensive and high-normal patients [9,10]. Many non-clinical studies give evidence that forest recreation for the purpose of health improvement may have a salutary influence on participants from many countries. Studies conducted in Japan showed that staying in a forest environment reduced negative symptoms of stress [11], induced cardiovascular relaxation [12], and had an impact on physiological and psychological indices [13,14,15,16,17]. Studies from Taiwan also reported that this intervention may be effective in stress reduction [18], as did studies conducted in South Korea [19,20] and in Denmark [6]. The above examples indicate that forest recreation is an effective remedy for many health problems in many countries and could be considered as a possible additional therapy for mental diseases. Furthermore, other research has suggested that forest recreation may also cause immunological stimulation or increase the number of cells involved in the body’s response to cancer [21,22,23,24]. It is worth mentioning that also other forms of nature therapy are effective [25].

Many people in the world are affected by mental health problems [26]. This is costly and harmful for societies and thus, interventions in this area are greatly needed. The development of additional forms of therapy is therefore important. Based on this knowledge, it is necessary to assess the effectuality of forest recreation on patients in mental hospitals. Previous studies [27] that have confirmed the positive influence of forest recreation on indices of the physical and psychological health of psychiatric inpatients suggest that negative symptoms of mental diseases may be reduced by such therapy. If this is so, this activity could be helpful as an additional therapy in treating some mental health problems, such as anxiety and depression. Alternatively, staying in an environment not appropriate for forest therapy, such as a forest without a view and with a dense understory, could induce fear [28], which is not a desired effect. For this reason, it is unclear whether or not forest recreation will induce a therapeutic effect in mental hospital patients.

To determine if forest recreation can induce positive effects on mood and anxiety levels, a pre-test–post-test design was used to assess the healing effect in two groups of patients: those with affective disorders and those with psychotic disorders. A recreational walk in a suburban forest near the mental hospital in the city of Olsztyn was applied as a form of forest therapy intervention. Appropriate psychological questionnaires, the Profile of Mood States (POMS) and State-Trait Anxiety Inventory (STAI), were administrated to assess any resulting health improvements. The results of comparisons between pre- and post-test are herein described and discussed, and conclusions for forest owners, foresters and therapists are also given.

The following research hypotheses were made in the study. For both groups of patients, with affective disorders and with psychotic disorders:

- forest therapy will have a positive effect on mood (on the POMS scale subscales),
- forest therapy will have a positive effect on anxiety (STAI-S values).

## 2. Materials and Methods

### 2.1. Participants

Fifty patients from the Provincial Unit of Psychiatric Treatment in Olsztyn voluntarily participated in this study. Patients were in the day-care hospital ward for reported mental health problems confirmed by stuff. During a few-day stay in the hospital, the patients took medicines that were appropriate to their medical conditions. The mean age of patients was 42.44 years (±13.23 SD); 27 were female and 23 were male.

One of many therapeutic activities during these stays was participation in forest recreation in the nearest suburban forest. Some patients could not attend the therapy due to poor health; for example, patients with severe depression did not want to participate and those suffering hallucinations were excluded. To balance the need to quickly perform the tests and the need to obtain reliable results, an optimal sample size was used. Previous research indicates that a sample size of 12–16 participants is sufficient to draw significant conclusions in forest therapy experiments [18,28], and thus the groups of 23 patients with psychotic disorders and 27 patients with affective disorders included in this study are large enough to provide valuable information.

Patients were recruited according to strict criteria. Two factors determined the inclusion in the study: belonging to one of the two disease criteria (patients with psychotic or affective disorders were selected) and willingness to participate in the study. Participants were qualified for the examination by a physician. Gender and age were not criteria for discrimination from the research. Patients were asked for their willingness to participate in the study by the physician and a consent form had to be obtained. The following inclusion and exclusion criteria were followed when selecting patients for the study.

Criteria for inclusion:

- at least two weeks of hospitalisation in a psychiatric ward
- psychotic disorders (mental disorders that cause abnormal thinking and perceptions, in this case: schizophrenia) diagnosed by a specialist in the field of psychiatry (F20–F29) or affective disorders (F30–F39) according to the International Classification of Diseases (ICD-10)
- consent to participate in the study.

Exclusion criteria:

- mental state that makes it impossible to leave the psychiatric ward
- movement disorders or other somatic diseases that prevent participation in the study.

### 2.2. Study site

**Figure 1:**
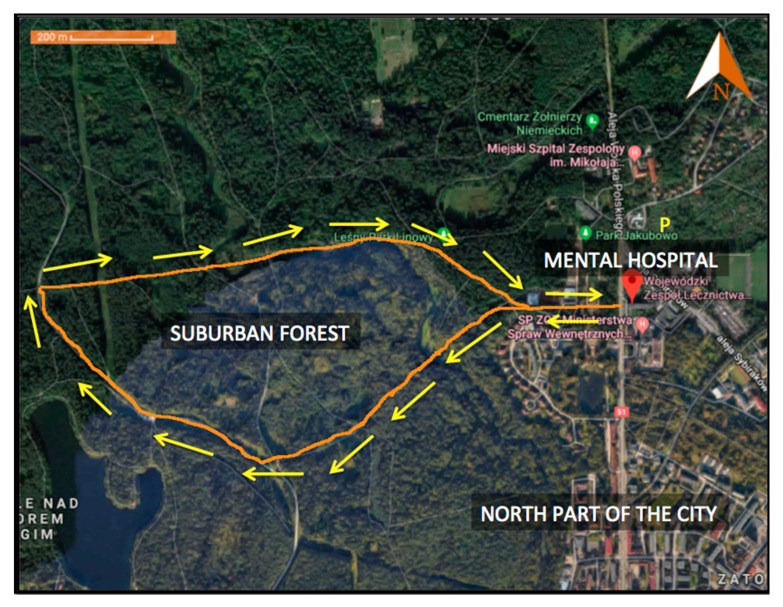
The route walked during a typical forest recreation programme at the Provincial Unit of Psychiatric Treatment in Olsztyn, Poland.

The Provincial Unit of Psychiatric Treatment, which includes a mental hospital in its complex, is located on the northern outskirts of the city of Olsztyn, in north-eastern Poland (GPS 20°30’E 53°47’N). The hospital is in a part of the city that borders a suburban forest (Figure 1). The climate in Olsztyn is temperate, mean annual temperature is 7.9 °C, mean annual precipitation is 635 mm and altitude is 139 m. The weather during days of forest recreation was fine, with an approximate temperature of 20–25 °C, without strong wind and without precipitation.

The area of the suburban forest is covered mainly by 65- to 180-year-old Scots pine (*Pinus sylvestris* L.), with some 95- to 105-year-old Norway spruce (*Picea abies* (L.) H. Karst.) and 95- to 110-year-old pedunculate oak (*Quercus robur* L.), and the occasional 15-year-old common beech (*Fagus sylvatica* L.). Ground in this part of the suburban forest is covered with moss and herbaceous vegetation. All views in the place selected for forest therapy showed forest, mainly undisturbed by buildings or other objects.

### 2.3. Procedure

Between August and September of 2018, patients of the mental hospital in Olsztyn participated voluntarily in forest recreation interventions organised by medical staff at the hospital. On 12 different occasions throughout the day (four to five patients per one forest therapy session), patients were encouraged to participate in forest walks with additional exercises in the forest environment (walking, stretching, watching landscapes). This intervention took place under the supervision of a qualified therapist. Patients spent an hour and forty-five minutes in the forest during each forest recreation programme. Patients participated in the therapy once, each time the therapy was organized around noon. The first small group participated in the therapy on July 27th and the last group on November 29th. The number of patients in the hospital ward was small, so they could not communicate and exchange experiences about the experiment at the time (patients who participated in the study stayed in the hospital for a short time, hence they could not contact new patients and tell them about the therapy). The walking route for forest recreation is shown in Figure 1. Before and after interventions, psychological questionnaires were administrated to patients, allowing them to assess their perceived feelings before and after the forest therapy. The questionnaires before and after interventions were filled out in an indoor environment, in conference rooms in the hospital.

### 2.4. Measurements

To measure the response of patients to the forest recreation intervention, two psychological questionnaires were administrated before and after the intervention.

To assess the effect of forest therapy on emotional state, the Polish 65-item version of the Profile of Mood States (POMS) questionnaire was chosen [29,30]. The POMS is a reliable and valid instrument for assessing psychological distress [31], and has been used previously to estimate the influence of a forest environment on mood states [32,33]. This questionnaire measures six mood states: confusion, fatigue, anger–hostility, tension–anxiety, depression–dejection and vigour. A five-point Likert scale was used for each item to evaluate participants’ mood states, with each item assessed from 0 (strongly agree) to 4 (strongly disagree).

To measure the effect of forest recreation on levels of anxiety, the State-Trait Anxiety Inventory (STAI) was used [34]. The original STAI questionnaire is composed of two parts (STAI-S and -T). The STAI-S is meant to measure the level of anxiety in the present moment (20 items, state anxiety) and the STAI-T is meant to measure anxiety levels as a personal characteristic (another 20 items). For this research, the former was most appropriate and thus the Polish 20-item STAI-S was applied [35]. A four-point Likert scale, ranging from (1) strongly disagrees to (4) strongly agree, was used to evaluate patients’ anxiety levels.

Both scales used are reliable and have been tested in terms of their usefulness in research regarding forest therapy. Anxiety measurement in psychiatric hospital patients is very important, which is why we chose to also apply the STAI-S questionnaire, despite the tension–anxiety subscale already included in the POMS.

### 2.5. Data Analysis

All data were stored in Excel (Microsoft, Redmont, WA, USA), and mean values and standard deviation (SD) values were also calculated using this programme. All further analysis was conducted using SPSS Statistics Version 24 (IBM, Armonk, NY, USA). For comparison between pre-test and post-test measurements, a paired sample *t*-test was applied and the Holm correction was used to adjust *p* values. The effect size (ES) with Cohen’s d was also calculated. An ES value of approximately 0.2 was described as a small effect, approximately 0.5 as a medium effect and approximately 0.8 as a large effect.

## 3. Results

### 3.1. Age and gender distribution

The study involved 18 women (average age = 44.88) with affective disorders and 9 women (average age = 39.77) with psychotic disorders. Nine men (average age = 44.44) with affective disorders and 14 men (average age = 39.71) with psychotic disorders also participated in the study.

### 3.2. Patients with Affective Disorders

Results of the paired sample *t*-test examining the psychological differences for patients with affective disorders before (pre-test) and after (post-test) the forest recreation programme are presented in Table 1. Following the forest recreation programme, there was a significant decline in four negative mood states of the POMS scale: tension–anxiety (*t* = 4.51, *p* < 0.001), depression–dejection (*t* = 6.42, *p* < 0.001), fatigue (*t* = 3.23, *p* = 0.006) and confusion (*t* = 8.82, *p* < 0.001). Furthermore, there was a significant increase in vigour levels post-test in comparison to those levels before the test (*t* = −4.35, *p* = 0.001). Regarding anxiety, patient levels showed a significant decrease post-test (*t* = 4.88, *p* < 0.001). The level of the anger–hostility mood state did not change after the forest recreation programme (*t* = 0.52, *p* = 0.605). In addition, in patients with affective disorders, the size of the effect was greatest for two mood states of the POMS scale, confusion (ES = 3.46) and depression–dejection (ES = 2.51), meaning that these two indicators were the most responsive to change.

### 3.3. Patients with Psychotic Disorders

Results of the paired sample *t*-test regarding the psychological differences for patients with psychotic disorders pre- and post-test are presented in Table 2. After the programme, there was a significant decrease in three negative mood states of the POMS scale: tension–anxiety (*t* = 3.04, *p* = 0.018), depression–dejection (*t* = 3.44, *p* = 0.009), confusion (*t* = 4.72, *p* = 0.001) and anger-hostility (*t* = 2.57, *p* = 0.035), and also STAI-S level significantly decreased (*t* = 5.68, *p* < 0.001). There was also a significant increase in one positive mood state of POMS, vigour (*t* = 5.78, *p* < 0.000). Anxiety levels in patients with psychotic disorders decreased significantly after the forest recreation programme (*t* = 5.68, *p* < 0.001). The level of the mood state fatigue did not change under the influence of the forest recreation programme. The size of the effect was greatest for vigour (ES = 2.46) and tension–anxiety (ES = 2.42), indicating that these two characteristics were most affected by the forest recreation programme.

## 4. Discussion

### 4.1. Patients with Affective Disorders

This study indicated that a programme of forest recreation lasting one hour and forty-five minutes has had a positive influence on the psychological health of patients with affective disorders, which confirms other studies [2,3,4,5,6,8,9]. This intervention worked as healing therapy, with patients reporting significantly lower levels of four negative aspects of mood measured by the POMS questionnaire: tension–anxiety, depression–dejection, fatigue and confusion. Only one negative aspect of mood, anger–hostility, showed no significant change between pre- and post-test. Vigour, an indicator of positive mood, increased significantly after the intervention. Anxiety levels, measured using the STAI-S questionnaire, significantly decreased. These findings are consistent with studies that tested the effect of forest therapy on healthy Polish young adults [31,36] and found that some indicators of negative mood decreased after exposure to a forest environment. These findings are in opposition to those described in the work of Bielinis et al. [31], in which healthy Polish young adults were tested and only one negative mood indicator, fatigue, increased significantly (mean pre-test = 1.61; mean post-test = 0.80). In the current study, fatigue actually decreased (mean pre-test = 1.59; mean post-test = 1.27), suggesting that the reactions of healthy adults and non-healthy adults may differ. In affective inpatients, perhaps fatigue is difficult to change via a forest recreation programme, whereas in healthy adults, it is more variable. In another study by Bielinis et al. [36], changes in fatigue levels of working or studying healthy young adults were only marginally nonsignificant (*p* = 0.084, large effect size) and decreased after exposure to a forest environment (mean pre-test = 1.22; mean post-test = 0.81), supporting this suggestion. In other studies, in which psychiatric inpatients were examined, no significant effects of forest therapy on mood states were found, but in these studies lower numbers of participants were tested (10 patients in experimental group and 10 in control group). In these studies, levels of cortisol and levels of depression measured using the Beck Depression Inventory were significantly lower in inpatients after forest therapy [7], indicating observable positive mental health changes.

Anxiety levels measured using the STAI-S questionnaire significantly decreased after the forest recreation programme (mean pre-test = 49.39, mean post-test = 38.57), but remained higher than those of healthy participants in a similar investigation (mean pre-test = 30.19, mean post-test = 25.44) [18]. These results may not be excellent, but they do provide good information for practitioners, as they indicate that an intervention of approximately one hour and forty-five minutes of forest recreation may occasionally decrease the anxiety of inpatients. This may provide a good background for additional psychotherapy, in which a lower level of anxiety is helpful. As other studies confirmed, forest therapy may be effective [2,4,5].

### 4.2. Patients with Psychotic Disorders

The one hour and forty-five minutes forest recreation programme significantly decreased four indicators of negative mood states: tension–anxiety, depression–dejection, anger–hostility and confusion. Fatigue, however, did not show significant change in response to the forest recreation programme. For patients with psychotic disorders with symptoms that include a high level of fatigue (pre-test means for psychotic patients = 1.47, affective patients = 1.59, healthy individuals = 1.22 [36]), this fatigue may not be easily reduced by any kind of additive therapy, suggesting that one symptom of schizophrenia, measured by fatigue, is likely difficult to change through forest therapy. Other important symptoms of schizophrenia, however, such as high levels of anxiety (pre-test means for psychotic patients = 1.76, affective patients = 1.58, healthy individuals = 0.90 [36]), may be significantly reduced in these patients, which is valuable information for practitioners. The level of anxiety measured using the STAI-S questionnaire was also lower (STAI-S score nearly 22% lower after forest therapy). Additionally, level of vigour increased after therapy (by 45%) and, in contrast to patients with affective disorders, anger–hostility significantly decreased. In other studies, the effect on psychotic symptoms was also observable [7].

Once the abovementioned parameters decreased or increased after therapy, the new levels were close to those of healthy individuals in other research [31]. The optimal levels of these psychological parameters are important for patient health and are negatively related to symptoms of schizophrenia, which may be ameliorated by a forest environment. In other research, some forms of physical activity decreased these negative symptoms [37]. This is related to forest therapy, because one of its elements is movement. Thus, forest therapy intervention may successfully stabilise the mood and anxiety levels of patients with psychotic disorders. This is valuable information for therapists, doctors and other practitioners, and forest therapy could perhaps be conducted as an occasionally, complementary therapy in psychosis, despite appearing counterintuitive. Before conducting forest therapy interventions, the authors of this research hypothesised that interactions between patients with psychosis and a forest environment might increase negative symptoms, but the opposite occurred here, as in other studies [7]. This is a positive outcome that should be tested in other experiments. Additionally, other physiological indices (e.g., fMRI scans, biomarkers examinations in the blood) should be measured to further investigate the real, physiological effects of forest therapy on patients with psychosis. Any further information concerning this potentially extraordinary therapy will be most useful, as it appears to effectively aid patients.

### 4.3. Limitations

There are some limitations to the study described in the article. Participants in the study were of different sexes, but this factor was not included in the analysis. This will be possible in future planned, randomized controlled studies. Another limitation in this work is the fact that in the study, the researchers did not record how much of a given medical drug a particular patient took, therefore, this factor could not be used as a covariate in the analysis. In future studies (this one can be considered as a pilot test), the authors will consider this factor in randomized trials. The other limitation is the quasi-experimental study design, without a planned control group (without control in which participants would, for example, only participate outside the hospital, but not in the forest, just like the experimental group). Thus, although pre- and post-effects were observed, whether they are due to expectation (e.g., placebo effect) or true effects could not be discerned. This problem should be resolved in further studies on this topic. The other limitation is the conducted analysis. There was no control for potential confounders. Thus, any effects observed could be due to confounding factors instead of treatment effects. This problem will be solved in subsequent experiments in which the level of additional variables will be measured and analyzed. Also, it would be interesting to check whether patients who show some kind of preferences for the forest environment will also achieve greater benefits from forest therapy (according to the logic: the better they like the forest, the better the forest works on them, according to the results of work, in which factors responsible for the prediction of the positive impact of garden therapy on the subjects were described [38]). Unfortunately, this study did not test this, which is another limitation, but it suggests direction of future activities for other scientists in this area. Another limitation is the fact that there was no control group in the study. This problem will be eliminated in future, randomized controlled trials. Unfortunately, in the study, the authors did not know exactly how many patients there were in the ward, so it was difficult to calculate what was the reliability of the sample in this study, so it was considered as one of the limitation. It is only known that strictly new patients were involved in the experiment, and those who did not want to participate remained in the ward.

## 5. Conclusions

In the case of a group of patients with:

(1) affective disorders
  - forest therapy had a positive effect on nearly all POMS scale subscales, with the exception of anger–hostility,
  - confusion and depression–dejection were significantly decreased,
  - the level of anxiety measured with the STAI-S scale significantly decreased.
(2) psychotic disorders
  - the confusion and vigour subscales and the STAI-S scale showed the greatest change,
  - in the case of the fatigue subscale, no significant changes were observed in patients with psychotic disorders.

The observed changes in psychological indicators in psychiatric hospital patients indicate that the intervention of forest therapy may positively affect their mental health. Varying reactions were also observed depending on the group of diseases a patient experienced. In the case of people with psychotic disorders, the greatest effect of therapy was observed regarding vigour, whereas in the case of patients with affective disorders, the largest reactions were observed in relation to the confusion and depression–dejection traits. Changes in psychological indicators are therefore appropriate to the characteristics of a given disorder. This is valuable information for therapists, doctors and other practitioners.

## Figures and Tables

**Table 1:** Effects of the forest recreation programme on mood states and anxiety of patients with affective disorders.

Psychological Indices (Affective Patients)	Pre-test	Post-test	*t*	*p*	Rate of Change (%)	ES
	Mean ± SD	Mean ± SD				
**Mood State (POMS)**						
Tension-anxiety	1.58 ± 0.75	1.05 ± 0.8	4.51	<0.000 ***	−33.85%	1.77
Depression-dejection	1.8 ± 0.86	1.11 ± 0.69	6.42	<0.000 ***	−38.05%	2.52
Anger-hostility	0.89 ± 0.43	0.85 ± 0.45	0.52	0.605	−4.86%	0.21
Fatigue	1.59 ± 0,8	1.27 ± 0.6	3.23	0.006 **	−20.27%	1.27
Confusion	1.77 ± 0.56	0.97 ± 0.56	8.82	<0.000 ***	−45.21%	3.46
Vigor	1.46 ± 0.75	2.05 ± 0.69	−4.35	0.001 **	40.64%	1.71
Anxiety (STAI-S)	50.26 ± 13.91	39.19 ± 9.41	4.88	<0.000 ***	−22.03%	1.91

Note: POMS: Profile of Mood States; STAI-S: The State-Trait Anxiety Inventory, State Anxiety; ES: Effect Size; ** *p* < 0.01, *** *p* < 0.001; Holm correction was applied; n = 27.

**Table 2:** Effects of the forest recreation programme on mood states and anxiety of patients with psychotic disorders.

Psychological Indices (Psychotic Patients)	Pre-Test	Post-Test	*t*	*p*	Rate of Change (%)	ES
	Mean ± SD	Mean ± SD				
**Mood State (POMS)**						
Tension-anxiety	1.76 ± 0.97	1.18 ± 0.57	3.04	0.018 *	−32.88%	1.3
Depression-dejection	1.46 ± 0.82	0.99 ± 0.52	3.44	0.009 **	−32.08%	1.47
Anger-hostility	1.19 ± 0.51	0.95 ± 0.38	2.57	0.035 *	−20.06%	1.1
Fatigue	1.47 ± 0.8	1.34 ± 0.52	0.85	0.404	−8.90%	0.36
Confusion	1.69 ± 0.71	0.98 ± 0.53	4.72	0.001 **	−41.91%	2.01
Vigor	1.53 ± 0.59	2.22 ± 0.45	−5.78	<0.000 ***	45.04%	2.46
Anxiety (STAI-S)	49.39 ± 9.08	38.57 ± 6.56	5.68	<0.000 ***	−21.91%	2.42

Note: POMS: Profile of Mood States; STAI-S: The State-Trait Anxiety Inventory, State Anxiety; ES: Effect Size; * *p* < 0.05. ** *p* < 0.01. *** *p* < 0.001; Holm correction was applied; n = 23.
